# Cerebrospinal fluid biomarkers in patients with idiopathic normal pressure hydrocephalus with temporary response to shunt insertion

**DOI:** 10.1186/2045-8118-12-S1-P7

**Published:** 2015-09-18

**Authors:** Huan Wee Chan, Patricia Anne Haylock-Vize, Edward William Dyson, Aswin Chari, Claudia Craven, Samir A Matloob, Neekhil A Patel, Simon D Thompson, Syed N Shah, Andrew R Stevens, Jinendra Ekanayake, Ahmed K Toma, Laurence Dale Watkins

**Affiliations:** 1 The National Hospital for Neurology and Neurosurgery, UK

## Introduction

Obstruction to cerebrospinal fluid (CSF) flow in idiopathic normal pressure hydrocephalus (iNPH) results in reduced CSF total tau (t-tau) and amyloid-β 42 (Aβ42) protein concentrations [1]. Restoration of normal CSF flow dynamics with ventriculoperitoneal (VP) shunt allows these biomarkers to clear from extracellular fluid into the CSF(1). CSF biomarkers in iNPH have been an interesting subject with initials results suggestive of reduced t-tau and amyloid-β. A subgroup of probable iNPH patients responds favorably to VP shunt insertion but for a brief period (temporary responders). In our unit, these patients are further investigated with assessment of the effect of shunt tapping on walking speed. A large proportion underwent shunt revision. In this population, CSF biomarkers were studied over a prolonged period of time.

## Methods

We included 5 patients (4 females; 1 male; mean age 77 years; age range 68-94 years) with temporary shunt responsive iNPH in the study. CSF t-tau and Aβ42 protein levels were measured at the time of lumbar drainage prior to initial shunt insertion, during shunt insertion, shunt tap to investigate shunt function and finally at shunt revision. The changes in these biomarkers were calculated.

## Results

The duration between the first sample and the last sample at shunt revision was 527.8 ± 99.7 days (mean±SD). The mean t-tau levels were 181.7pg/ml, 821.0pg/ml, 392.5pg/ml and 492.25pg/ml at the time of lumbar drainage, during first shunt insertion, shunt tap and during shunt revision, respectively. The corresponding mean Aβ42 levels were 465.0pg/ml, 407.8pg/ml, 646.75pg/ml and 472.25pg/ml, respectively.

## Conclusions

Our results demonstrated features suggestive of mild degenerative pathology distinct from Alzheimer's pattern. It remains to be proven if CSF drainage alters the biomarkers levels.
